# First identification of *Microsporidia MB* in *Anopheles coluzzii* from Zinder City, Niger

**DOI:** 10.1186/s13071-023-06059-7

**Published:** 2024-01-29

**Authors:** Lamine Mahaman Moustapha, Illiassou Mamane Sadou, Ibrahima Issa Arzika, Laminou Ibrahim Maman, Michel K. Gomgnimbou, Maurice Konkobo, Abdoulaye Diabate, Etienne Bilgo

**Affiliations:** 1 Faculté de Sciences et Techniques de l’Université André Salifou, Zinder, Niger; 2 Centre de Recherche Médicale et Sanitaire (CERMES), Niamey, Niger; 3 Centre d’Excellence Africain en Innovations Biotechnologiques pour l’Elimination des Maladies à Transmission Vectorielle (CEA/ITECH-MTV), Université Nazi Boni (UNB), Bobo Dioulasso, Burkina Faso; 4 Centre Muraz, Institut National de Santé Publique (INSP), Bobo Dioulasso, Burkina Faso; 5 Institut de Recherche en Sciences de la Santé (IRSS), Direction Régionale de l’Ouest, Bobo Dioulasso, Burkina Faso

**Keywords:** Malaria, *Plasmodium falciparum*, *Anopheles gambiae* complex, *Microsporidia MB*, Niger

## Abstract

**Background:**

Malaria, a disease transmitted by *Anopheles* mosquitoes, is a major public health problem causing millions of deaths worldwide, mostly among children under the age of 5 years. Biotechnological interventions targeting parasite-vector interactions have shown that the microsporidian symbiont *Microsporidia MB* has the potential to disrupt and block *Plasmodium* transmission.

**Methods:**

A prospective cross-sectional survey was conducted in Zinder City (Zinder), Niger, from August to September 2022, using the CDC light trap technique to collect adult mosquitoes belonging to the *Anopheles gambiae* complex. The survey focused on collecting mosquitoes from three neighborhoods of Zinder (Birni, Kangna and Garin Malan, located in communes I, II and IV, respectively). Collected mosquitoes were sorted and preserved in 70% ethanol. PCR was used to identify host species and detect the presence of *Microsporidia MB* and *Plasmodium falciparum* infection.

**Results:**

Of the 257 *Anopheles* mosquitoes collected and identified by PCR, *Anopheles coluzzii* was the most prevalent species, accounting for 97.7% of the total. *Microsporidia MB* was exclusively detected in *A. coluzzii*, with a prevalence of 6.8% (17/251) among the samples. No significant difference in prevalence was found among the three neighborhoods. Only one *An. coluzzii* mosquito tested PCR-positive for *P. falciparum*.

**Conclusions:**

The results confirm the presence of *Microsporidia MB* in *Anopheles* mosquitoes in Zinder, Niger, indicating its potential use as a biotechnological intervention against malaria transmission. However, further studies are needed to determine the efficacy of *Microsporidia MB* to disrupt *Plasmodium* transmission as well as its impact on vector fitness.

**Graphical Abstract:**

**Figure:**
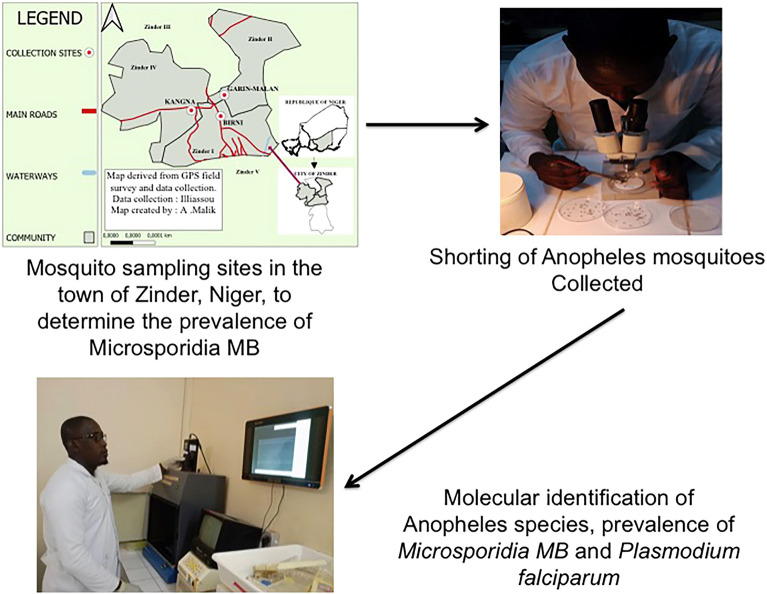

**Supplementary Information:**

The online version contains supplementary material available at 10.1186/s13071-023-06059-7.

## Background

Malaria is a disease caused by *Plasmodium* parasites transmitted to humans through the bite of female mosquitoes of the genus *Anopheles*. According to the latest WHO report, there were approximately 247 million cases of malaria and 619,000 deaths due to malaria worldwide in 2021 [1]. The WHO African region continues to bear the brunt of malaria, accounting for 95% of all malaria cases and 96% of all malaria deaths worldwide in 2021, which translates to 228 million cases and 602,000 deaths, respectively [1]. In this region, 80% of deaths involve children under the age of 5 years. In Niger, malaria is a major public health problem, with 4.1 million cases of malaria and 5056 associated deaths in 2021 in this country alone [2]. Malaria is transmitted by a wide range of *Anopheles* species with different ecological ranges and varying efficiencies in *Plasmodium* genus transmission. The main vector involved in malaria transmission in southern Niger, including Zinder City, is *Anopheles coluzzii* [3, 4]. The large-scale campaigns to distribute insecticide-treated mosquito nets over the past 15 years in Niger have reduced malaria cases by about 40% [1]. However, the efficacy of this chemical-based malaria prevention strategy is currently being challenged by the major problem of mosquito resistance to various classes of commonly used insecticides [5]. The WHO has reported that progress has indeed come to a standstill, with the global incidence of malaria having remained essentially unchanged between 2014 and 2016 [1]. Increasing emphasis is being placed on alternative strategies, including biotechnological strategies for disease control. It is important to explore new ways beyond the conventional methods used so far against malaria vectors through the innovative application of microbiology-based interventions targeting specific aspects of the process of parasite-vector interaction, thereby leading to disease prevention rather than simply reducing mosquito populations alone [6]. Consequently, increasing efforts are being focusing on the search for natural symbionts associated with mosquitoes that are capable of reducing vector competence.

In this context, a microscopic fungus of the genus *Microsporidia*, is attracting the interest of researchers. *Microsporidia* is a group of obligate intracellular parasites that have undergone severe selective reduction over time [7]. They are widespread in nature and can be found as parasites in all classes of vertebrates, including humans and most invertebrates. These organisms present a wide range of developmental cycles but are generally divided into two broad categories: monomorphic forms such as Nosema and true polymorphic forms presenting a complex life-cycle involving elements of both sexual and asexual reproduction. In addition, multiple types of spores can be present at various stages within the host. Some strains of *Microsporidia* are present as symbionts in mosquito populations, including malaria vectors. These types of symbionts can be used to combat vector mosquitoes or impact their vector competence. It has recently been demonstrated in Kenya that *Microsprodia MB* disrupted and blocked* Plasmodium* transmission in *Anopheles arabiensis* [8]. In Ghana, Akorli et al. [9] showed that *Microsporidia MB* is mostly associated with *Anopheles gambiae* sensu stricto (*An. gambiae* s.s.) and *An. coluzzii*. Unlike other *Microsporidia* associated with mosquitoes, *Microsporidia MB* confers no significant negative effects on host fertility, fecundity, development and longevity [8, 9]. These characteristics make *Microsporidia MB* an attractive candidate for the control and transmission of *Plasmodium* by *Anopheles* [6].

To the best of our knowledge, there is currently no available data in Niger concerning the presence of *Microsporidia MB* and its distribution among major malaria vectors. The objective of this study was to examine the occurrence of *Microsporidia MB* in mosquitoes belonging to the *An. gambiae* complex in the urban area of Zinder City (Zinder), Niger.

## Methods

### Study setting and mosquito sampling methods

The study was conducted in Zinder, also known as Damagaram, which is located in the Sahelian zone in southeastern Niger, approximately 900 km from the capital city of Niamey. It is characterized by a tropical semi-arid climate with two seasons: the rainy season from June to September and the dry season from October to February, with two temperature periods (cold and hot). The city experiences extreme seasonal variations in perceived humidity and temperature, with an average annual rainfall of 450 mm and temperatures ranging from 25.8 °C to 32.4 °C.

**Fig. 1:**
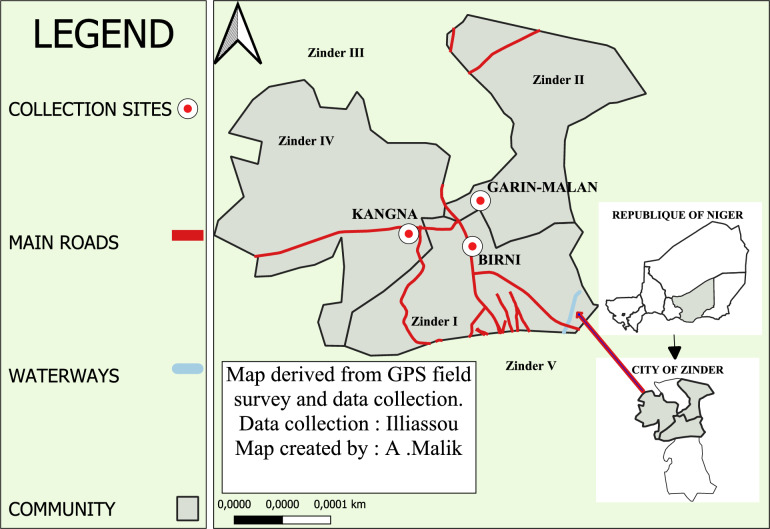
Mosquito sampling sites in the city of Zinder, Niger, for determining the prevalence of *Microsporidia MB*

 This study was a prospective cross-sectional survey conducted during the rainy season from August to September 2022. The study focused on all mosquitoes from the *An. gambiae* complex collected from the Birni, Kangna and Garin Malan neighborhoods of Zinder, located in communes I, II, and IV, respectively (Fig. 1). The biological sample for this study comprised adult mosquitoes from the *An. gambiae* complex. The CDC light trap technique was used to collect mosquitoes. The head of each household provided informed consent before the light trap was installed in the home. Houses for collection were chosen at random. The traps were placed in each home in the evening at 6 p.m. and retrieved the next morning at 7 a.m. The number of mosquitoes captured in each house was determined, and each house was assigned a number. The captured insects were first anesthetized by cold and then placed on a petri dish under a binocular microscope at ×40 magnification for sorting of *Anopheles* individuals using an identification key. The contents of each trap were then divided into batches of 10–20 *Anopheles* in numbered cryotubes and preserved in 70% ethanol.

### Molecular identification of mosquito species and detection of *Microsporidia MB* and *Plasmodium falciparum*

The collected mosquitoes were initially morphologically identified and counted using the key provided by Coetzee [10]. DNA from individual whole mosquito bodies was extracted using a 2% Cetyltrimethyl ammonium bromide (CTAB) extraction protocol [11] for the molecular determination of mosquito species, the presence of *Microsporidia MB*, and *Plasmodium falciparum* infection. For each of these three molecular identifications, specific PCR reactions and programs were followed as described below. For all of these PCRs, PCR products were subsequently loaded onto a 2% agarose gel supplemented with ethidium bromide at 0.2 μg/ml and electrophoresed for 1 h at 120 V and 400 mA. PCR products were visualized under UV light at 250 nm using a gel reader to capture photographs

For mosquito species identification, primers were adapted from the study by Santolamazza et al. [11]. SINE 200 PCR was performed to identify different sibling species within the *Anopheles gambiae* complex: *An. coluzzii*, *An. gambiae* s.s., and *An. arabiensis*, using specific SINE 200 primers. Briefly, the final volume for one sample was 15 μL, composed of 2 μL DNA, 3 μL Master Mix (5x Solis biodyne FIREPol Master), 0.6 μL of 10 μM primers (0.3 μL for each primer), and 9.4 μL ultrapure water. The PCR cycling conditions consisted of an initial denaturation at 95 °C for 15 min; followed by 45 cycles of denaturation at 95 °C for 30 s, annealing at 58 °C for 45 s and extension at 72 °C for 45 s; with a final elongation at 72 °C for 5 min. The expected electrophoresis bands are 479 bp for *An. coluzzii*, 249 bp for *An. gambiae* s.s., and 229 bp for *An. arabiensis*.

To detect *Microsporidia MB*, protocol described by Herren et al [8] was followed. Genomic DNA samples from whole mosquitoes were analyzed using conventional PCR to amplify the 18S sequence of the ribosomal RNA (rRNA) gene using MB18SF: CGCCGGCCGTGAAAAATTTA and MB18SR: CCTTGGACGTGGGAGCTATC [8]. The final volume by sample was 10 μL, the composition of which is 2 μL DNA, 2 μL (5X Solis byiodyne FIREPol Master), 1μL of 5 pM primers (0.5 μL for each primer), and 5 μL ultrapure water. The PCR cycling conditions consisted of an initial denaturation at 95 °C for 15 min; followed by 35 cycles of denaturation at 95 °C for 30 s, annealing at 59 °C for 45 s and extension at 72 °C. The expected electrophoresis band is 500 bp for *Microsporidia MB* positive samples (Additional file 1: Figure S1).

The detection of *P. falciparum* consisted of the search for sporozoites by a classical PCR technique using a pair of primers (﻿Pf1: GGAATGTTATTGCTAACAC; Pf2: AATGAAGAGCTGTGTATC) that targeted the gene coding for the parasite-specific circumsporozoite protein (CSP) [*8*]. The volume for PCR reactional mix for a sample was 15 μL: of which is 2 μL DNA, 3.8 μL (5X Solis byiodyne FIREPol Master) and 1μL of 5 pM primers (0.5 μL for each primer). The PCR cycling conditions consisted of denaturation for 3 min at 94 °C; followed by 35 cycles of 30 s at 94 °C, 1 min 15 s at 56 °C and 1 min at 68 °C; with a final extension for 10 min at 68 °C. Expected PCR bands observed at 501 bp were considered to indicate positivity for sporozoites.

## Results and discussion

The aim of the present study was to identify *Microsporidia MB* specifically in the *An. gambiae* complex from Zinder City, Niger. Of the 257 mosquitoes analyzed, 251 were identified as *An. coluzzii* and six as *An*. *gambiae* (s.s.). On average, 6.7% of all analyzed mosquitoes were positive for *Microsporidia MB*, and all positive mosquitoes were *An*. *coluzzii* (6.8%) (Table 1). Only one mosquito was positive for *P. falciparum*, and this mosquito tested negative for *Microsporidia MB* (Table 1; Additional file 1: Figure S1). The identification of *Microsporidia MB* in the *An. gambiae* complex in Zinder provides evidence that *Microsporidia MB* is present in *An. coluzzii*, the predominant malaria vector species in Niger specifically and in West Africa in general [2, 3, 12], albeit at a low prevalence. This result is consistent with the low rates of *Microsporidia MB* found in *An. gambiae* s.s. and *An. coluzzii* in Ghana [9] and *Anopheles* *arabiensis* in Kenya [8], and extends its range from East to West Africa. In the study carried out by Akorli et al*.* in Ghana [9], archived mosquito DNA samples collected from the field as larvae or adults over a 5-year period were used. These researchers found that *Microsporidia MB* was more prevalent in emerged adults from field-collected larvae than in field-caught adults, suggesting that this microsporidian can be efficiently transmitted vertically from larvae to adults or horizontally among larvae. In the present study, we only used mosquitoes collected prospectively from the field as adults and, therefore, we cannot draw any conclusions on the mode of contamination of *Anopheles* mosquitoes by *Microsporidia MB*. In East Africa, *An. arabiensis* was reported to be the most dominant species [13]. In Kenya, Herren et al. found that the microsporidian symbiont *Microsporidia MB was* present at a moderate prevalence in geographically dispersed populations of *An. arabiensis* [8]. It has been suggested that the presence of these parasites in mosquito populations may have implications for malaria transmission, as the presence of *Microsporidia MB* may affect the susceptibility of mosquitoes to *Plasmodium* infection. As this symbiont impairs *Plasmodium* transmission but is non-virulent and vertically transmitted, it could be investigated as a strategy to limit malaria transmission in Niger and other malaria endemic countries. Furthermore, the heterogeneous distribution of *An. gambiae* s.s. and *An*. *coluzzii* in different geographic areas of Zinder confirms that *An. coluzzii* was the predominant species in this locality with a high prevalence rate, while *An*. *gambiae* s.s. was the least represented species. These results are consistent with those reported by study from Labbo et al*.* in Niger [4]. This distribution of species could be due to variable climatic conditions [12]. In the current study, mosquitoes were collected during the rainy season which is characterized by peak rainfall (August–September). It is possible that the prevalence of *Microsporidia MB* infection is influenced by environmental conditions, such as peak rainfall. Herren et al*.* found that the highest infection rates were observed 4–6 weeks after peak rainfall [8], suggesting that environmental factors, such as humidity or temperature, may play a role in the transmission of *Microsporidia MB*. Further research is therefore needed to understand the specific mechanisms by which environmental conditions influence the prevalence of *Microsporidia MB* in *Anopheles* species.

**Table 1:** Prevalence of *Plasmodium falciparum* and *Microsporidia MB* within different species of the *Anopheles gambiae* complex in Zinder, Niger

Prevalence	*Anopheles* species	Prevalence, % (*n*/*N*)^a^
*Anopheles* species prevalence	*An. coluzzii*	97.67% (251/257)
	*An. gambiae* s.s.	2.33% (6/257)
*Microsporidia MB* prevalence	*An. coluzzii*	6.77 (17/251)
	*An. gambiae* s.s.	0.00% (0/6)
*Plasmodium falciparum* prevalence	*An. coluzzii*	0.398 (½51)
	*An. gambiae* s.s.	0.00% (0/6)

*s.s.* Sensu stricto

^a^*n* represents the number of positive cases and *N* represents the total population analyzed

The presence of *P. falciparum* in mosquitoes was also tested for by PCR; only one mosquito analyzed by PCR was positive for this parasite (Table 1). However, this mosquito did not test positive for *Microsporidia MB*. Thus, the results do not allow us to draw conclusions on a possible correlation between the two infections in the wild mosquito population (Additional file 1: Figure S1). Howevr, Herren et al.'s earlier study established this correlation [8], demonstrating that *Microsporidia MB* collected from field-infected *An*. *arabiensis* tested negative for *P. falciparum* gametocytes and had no detectable sporozoites when experimentally infected with *P. falciparum.*

## Conclusions

This study provides the first evidence for the presence of *Microsporidia MB* in *An*. *coluzzii* mosquitoes in the city of Zinder, Niger. The results suggest that further research is needed to investigate the potential impact of *Microsporidia MB* on malaria transmission in this region. Additionally, the low prevalence rate of *Microsporidia MB* and the heterogeneous distribution of *An. gambiae* and *An. coluzzii* in different geographic areas of Zinder City highlight the need for further studies aimed at gaining a better understanding of the epidemiology of malaria transmission in this region. Such studies may be useful in designing and implementing effective malaria control strategies that take into account the ecological and environmental factors that influence the distribution and prevalence of *Microsporidia MB* in malaria vectors.

### Supplementary Information

**Additional file 1: Figure S1.** Molecular detection of *Microsporidia MB* in *Anopheles coluzzii* from Zinder City, Niger using 2% TAE agarose gel.

**Additional file 2: Table S1.** Raw data on mosquito species collected and prevalence of *Microsporidia MB* and *Plasmodium falciparum* from Zinder City, Niger.

## Data Availability

Data for all analyses in this article are provided in the supplementary file.
