# Supplementary material for: First identification of Microsporidia MB in Anopheles coluzzii from Zinder City, Niger
Source: Parasit Vectors. 2024 Jan 29;17:39. doi: 10.1186/s13071-023-06059-7 (PMC10826271; doi:10.1186/s13071-023-06059-7)
Supplement: Supplementary file 1 — Additional file 1: Figure S1. Molecular detection of Microsporidia MB in Anopheles coluzzii from Zinder City, Niger using 2% TAE agarose gel. [file 13071_2023_6059_MOESM1_ESM.pptx]

## Slide 1
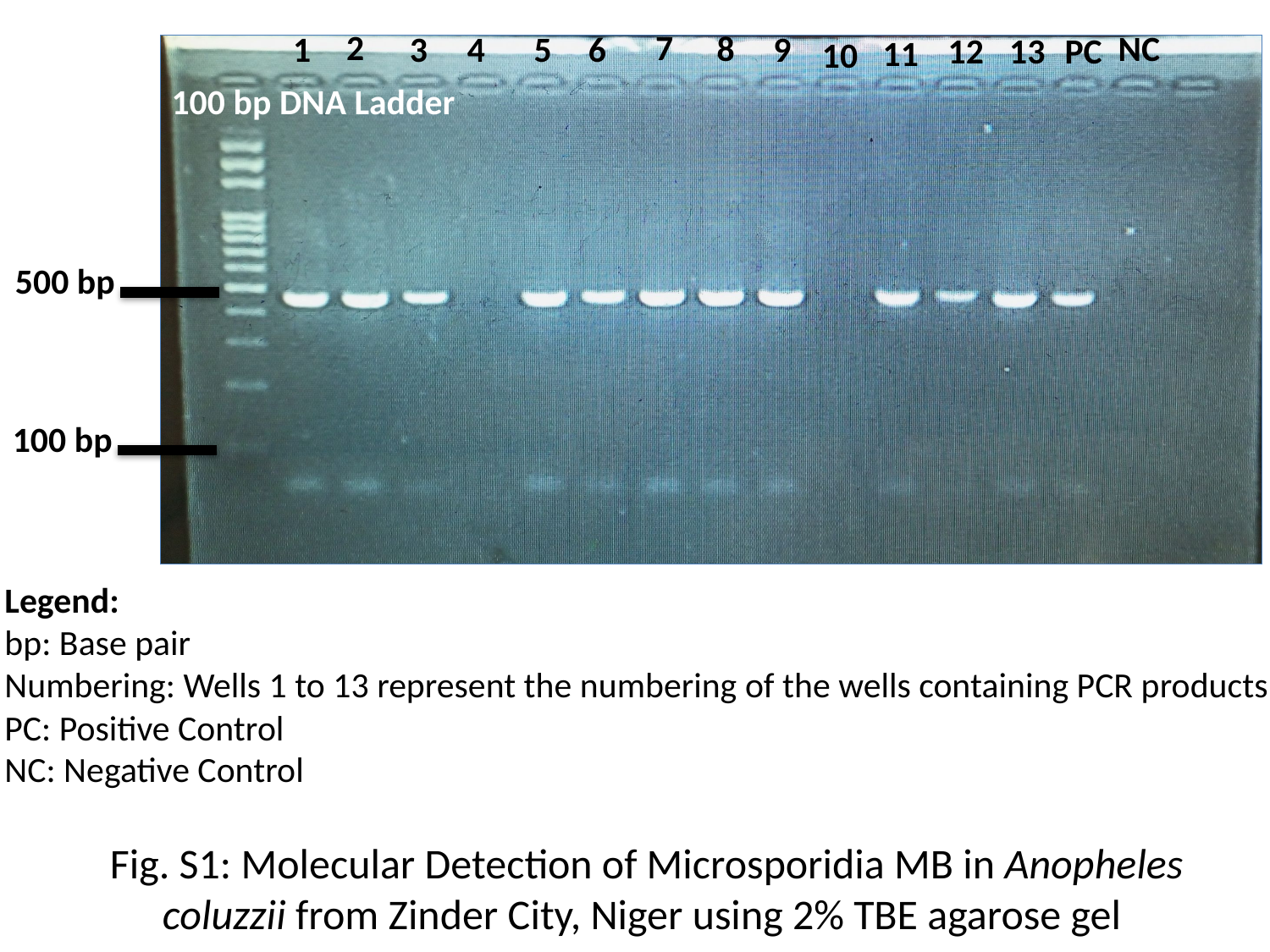

7
 2
 8
NC
4
 5
 6
 9
1
 3
12
13
PC
 11
 10
 100 bp DNA Ladder
500 bp
100 bp
Legend:
bp: Base pair
Numbering: Wells 1 to 13 represent the numbering of the wells containing PCR products
PC: Positive Control
NC: Negative Control
Fig. S1: Molecular Detection of Microsporidia MB in Anopheles coluzzii from Zinder City, Niger using 2% TBE agarose gel
