# Supplementary material for: First identification of Microsporidia MB in Anopheles coluzzii from Zinder City, Niger
Source: Parasit Vectors. 2024 Jan 29;17:39. doi: 10.1186/s13071-023-06059-7 (PMC10826271; doi:10.1186/s13071-023-06059-7)
Supplement: Supplementary file 2 — Additional file 2: Table S1. Raw data on mosquito species collected and prevalence of Microsporidia MB and Plasmodium falciparum from Zinder City, Niger. [file 13071_2023_6059_MOESM2_ESM.pdf]

Collection Sites	N° Samples	Anopheles_Specie	Microsporidian MB Status
KAGNAN	k1	An.coluzzii	negative
KAGNAN	k2	An.coluzzii	negative
KAGNAN	k3	An.coluzzii	negative
KAGNAN	k4	An.coluzzii	negative
KAGNAN	k5	An.coluzzii	negative
KAGNAN	k6	An.coluzzii	negative
KAGNAN	k7	An.coluzzii	negative
KAGNAN	k8	An.coluzzii	negative
KAGNAN	k9	An.coluzzii	negative
KAGNAN	k10	An.coluzzii	negative
KAGNAN	k11	An.coluzzii	negative
KAGNAN	k12	An.coluzzii	negative
KAGNAN	k13	An.coluzzii	negative
KAGNAN	k14	An.coluzzii	negative
KAGNAN	k15	An.coluzzii	negative
KAGNAN	k16	An.coluzzii	negative
KAGNAN	k17	An.coluzzii	negative
KAGNAN	k18	An.coluzzii	negative
KAGNAN	k19	An.coluzzii	negative
KAGNAN	k20	An.coluzzii	negative
KAGNAN	k21	An.coluzzii	negative
KAGNAN	k22	An.coluzzii	negative
KAGNAN	k23	An.coluzzii	negative
KAGNAN	k24	An.coluzzii	negative
KAGNAN	k25	An.gambiae	negative
KAGNAN	k26	An.coluzzii	negative
KAGNAN	k27	An.coluzzii	negative
KAGNAN	k28	An.coluzzii	positive
KAGNAN	k29	An.coluzzii	negative
KAGNAN	k30	An.coluzzii	negative
KAGNAN	k31	An.coluzzii	negative
KAGNAN	k32	An.coluzzii	negative
KAGNAN	k33	An.coluzzii	negative
KAGNAN	k34	An.coluzzii	negative
KAGNAN	k35	An.coluzzii	negative
KAGNAN	k36	An.coluzzii	negative
KAGNAN	k37	An.coluzzii	negative
KAGNAN	k38	An.coluzzii	positive
KAGNAN	k39	An.coluzzii	negative
KAGNAN	k40	An.coluzzii	negative
KAGNAN	k41	An.coluzzii	negative
KAGNAN	k42	An.coluzzii	negative
KAGNAN	k43	An.gambiae	negative
KAGNAN	k44	An.coluzzii	negative
KAGNAN	k45	An.coluzzii	negative
KAGNAN	k46	An.coluzzii	negative
KAGNAN	k47	An.coluzzii	negative
KAGNAN	k48	An.coluzzii	negative
KAGNAN	k49	An.coluzzii	negative

KAGNAN	k50	An.coluzzii	negative
KAGNAN	k51	An.coluzzii	negative
KAGNAN	k52	An.coluzzii	negative
KAGNAN	k53	An.coluzzii	negative
KAGNAN	k54	An.coluzzii	negative
KAGNAN	k55	An.coluzzii	negative
KAGNAN	k56	An.coluzzii	positive
KAGNAN	k57	An.coluzzii	negative
KAGNAN	k58	An.coluzzii	negative
KAGNAN	k59	An.coluzzii	negative
KAGNAN	k60	An.coluzzii	negative
KAGNAN	k61	An.coluzzii	negative
KAGNAN	k62	An.coluzzii	negative
KAGNAN	k63	An.coluzzii	negative
KAGNAN	k64	An.coluzzii	negative
KAGNAN	k65	An.coluzzii	negative
KAGNAN	k66	An.coluzzii	negative
KAGNAN	k67	An.coluzzii	negative
KAGNAN	k68	An.coluzzii	negative
KAGNAN	k69	An.coluzzii	negative
KAGNAN	k71	An.coluzzii	positive
KAGNAN	k71	An.coluzzii	negative
KAGNAN	k72	An.coluzzii	negative
KAGNAN	k73	An.coluzzii	negative
KAGNAN	k74	An.coluzzii	negative
KAGNAN	k76	An.coluzzii	negative
KAGNAN	k77	An.coluzzii	negative
KAGNAN	k78	An.coluzzii	negative
KAGNAN	k79	An.coluzzii	negative
KAGNAN	k80	An.coluzzii	negative
KAGNAN	k81	An.coluzzii	negative
KAGNAN	k83	An.coluzzii	negative
KAGNAN	k84	An.coluzzii	negative
KAGNAN	k86	An.coluzzii	negative
KAGNAN	k88	An.coluzzii	negative
KAGNAN	k89	An.coluzzii	negative
KAGNAN	K90	An.coluzzii	negative
KAGNAN	K91	An.coluzzii	negative
KAGNAN	K92	An.coluzzii	negative
KAGNAN	K93	An.coluzzii	negative
KAGNAN	K94	An.coluzzii	negative
KAGNAN	K95	An.coluzzii	negative
KAGNAN	K97	An.coluzzii	negative
KAGNAN	K98	An.coluzzii	negative
BIRNI	B1	An.coluzzii	negative
BIRNI	B7	An.coluzzii	positive
BIRNI	B8	An.coluzzii	negative
BIRNI	B9	An.coluzzii	negative
BIRNI	B11	An.coluzzii	negative
BIRNI	B12	An.coluzzii	negative

BIRNI	B13	An.coluzzii	negative
BIRNI	B14	An.coluzzii	negative
BIRNI	B15	An.coluzzii	positive
BIRNI	B17	An.coluzzii	negative
BIRNI	B19	An.coluzzii	negative
BIRNI	B21	An.coluzzii	positive
BIRNI	B22	An.coluzzii	negative
BIRNI	B23	An.coluzzii	negative
BIRNI	B24	An.coluzzii	negative
BIRNI	B25	An.coluzzii	negative
BIRNI	B26	An.coluzzii	negative
BIRNI	B27	An.coluzzii	positive
BIRNI	B28	An.coluzzii	negative
BIRNI	B29	An.coluzzii	negative
BIRNI	B30	An.coluzzii	negative
BIRNI	B31	An.coluzzii	negative
BIRNI	B34	An.coluzzii	positive
BIRNI	B35	An.coluzzii	negative
BIRNI	B36	An.coluzzii	negative
BIRNI	B37	An.coluzzii	negative
BIRNI	B39	An.coluzzii	negative
BIRNI	B41	An.gambiae	negative
BIRNI	B42	An.coluzzii	negative
BIRNI	B43	An.coluzzii	negative
BIRNI	B47	An.coluzzii	positive
BIRNI	B48	An.coluzzii	negative
BIRNI	B49	An.coluzzii	negative
BIRNI	B50	An.coluzzii	negative
BIRNI	B52	An.coluzzii	negative
BIRNI	B54	An.coluzzii	negative
BIRNI	B55	An.coluzzii	negative
BIRNI	B56	An.coluzzii	negative
BIRNI	B57	An.coluzzii	negative
BIRNI	B59	An.coluzzii	negative
BIRNI	B63	An.coluzzii	negative
BIRNI	B64	An.coluzzii	negative
BIRNI	B65	An.coluzzii	negative
BIRNI	B66	An.coluzzii	negative
BIRNI	B67	An.coluzzii	negative
BIRNI	B68	An.coluzzii	negative
BIRNI	B70	An.coluzzii	negative
BIRNI	B71	An.coluzzii	negative
BIRNI	B72	An.coluzzii	negative
BIRNI	B75	An.coluzzii	negative
BIRNI	B76	An.coluzzii	negative
BIRNI	B77	An.coluzzii	negative
BIRNI	B78	An.coluzzii	negative
GARIN_MALAN	GM3	An.coluzzii	negative
GARIN_MALAN	GM5	An.coluzzii	negative
GARIN_MALAN	GM6	An.coluzzii	negative

GARIN_MALAN	GM7	An.gambiae	negative
GARIN_MALAN	GM8	An.coluzzii	negative
GARIN_MALAN	GM9	An.coluzzii	negative
GARIN_MALAN	GM10	An.coluzzii	negative
GARIN_MALAN	GM11	An.coluzzii	negative
GARIN_MALAN	GM12	An.coluzzii	negative
GARIN_MALAN	GM13	An.coluzzii	negative
GARIN_MALAN	GM14	An.coluzzii	negative
GARIN_MALAN	GM15	An.coluzzii	negative
GARIN_MALAN	GM16	An.coluzzii	positive
GARIN_MALAN	GM19	An.coluzzii	negative
GARIN_MALAN	GM20	An.coluzzii	negative
GARIN_MALAN	GM21	An.coluzzii	negative
GARIN_MALAN	GM22	An.coluzzii	negative
GARIN_MALAN	GM23	An.coluzzii	negative
GARIN_MALAN	GM24	An.coluzzii	negative
GARIN_MALAN	GM4	An.coluzzii	negative
GARIN_MALAN	GM25	An.coluzzii	negative
GARIN_MALAN	GM26	An.coluzzii	negative
GARIN_MALAN	GM27	An.coluzzii	negative
GARIN_MALAN	GM28	An.coluzzii	negative
GARIN_MALAN	GM29	An.coluzzii	negative
GARIN_MALAN	GM30	An.coluzzii	negative
GARIN_MALAN	GM31	An.coluzzii	negative
GARIN_MALAN	GM32	An.coluzzii	negative
GARIN_MALAN	GM33	An.coluzzii	negative
GARIN_MALAN	GM35	An.coluzzii	negative
GARIN_MALAN	GM36	An.coluzzii	negative
GARIN_MALAN	GM37	An.coluzzii	positive
GARIN_MALAN	GM38	An.coluzzii	negative
GARIN_MALAN	GM39	An.coluzzii	negative
GARIN_MALAN	GM40	An.coluzzii	negative
GARIN_MALAN	GM41	An.coluzzii	negative
GARIN_MALAN	GM42	An.coluzzii	negative
GARIN_MALAN	GM43	An.coluzzii	negative
GARIN_MALAN	GM44	An.coluzzii	negative
GARIN_MALAN	GM45	An.coluzzii	negative
GARIN_MALAN	GM46	An.coluzzii	negative
GARIN_MALAN	GM47	An.coluzzii	negative
GARIN_MALAN	GM48	An.coluzzii	negative
GARIN_MALAN	GM49	An.coluzzii	negative
GARIN_MALAN	GM50	An.coluzzii	negative
GARIN_MALAN	GM51	An.coluzzii	negative
GARIN_MALAN	GM52	An.coluzzii	negative
GARIN_MALAN	GM53	An.coluzzii	negative
GARIN_MALAN	GM54	An.coluzzii	negative
GARIN_MALAN	GM55	An.coluzzii	negative
GARIN_MALAN	GM56	An.coluzzii	negative
GARIN_MALAN	GM57	An.coluzzii	negative
GARIN_MALAN	GM58	An.coluzzii	negative

GARIN_MALAN	GM59	An.coluzzii	negative
GARIN_MALAN	GM60	An.coluzzii	negative
GARIN_MALAN	GM61	An.coluzzii	negative
GARIN_MALAN	GM62	An.coluzzii	negative
GARIN_MALAN	GM63	An.coluzzii	negative
GARIN_MALAN	GM94	An.coluzzii	negative
GARIN_MALAN	GM65	An.coluzzii	negative
GARIN_MALAN	GM67	An.coluzzii	negative
GARIN_MALAN	GM69	An.coluzzii	negative
GARIN_MALAN	GM70	An.coluzzii	negative
GARIN_MALAN	GM71	An.coluzzii	negative
GARIN_MALAN	GM72	An.coluzzii	negative
GARIN_MALAN	GM73	An.coluzzii	negative
GARIN_MALAN	GM74	An.coluzzii	negative
GARIN_MALAN	GM76	An.coluzzii	negative
GARIN_MALAN	GM77	An.coluzzii	negative
GARIN_MALAN	GM78	An.coluzzii	negative
GARIN_MALAN	GM79	An.gambiae	negative
GARIN_MALAN	GM80	An.coluzzii	negative
GARIN_MALAN	GM81	An.coluzzii	positive
GARIN_MALAN	GM82	An.coluzzii	negative
GARIN_MALAN	GM83	An.coluzzii	negative
GARIN_MALAN	GM84	An.coluzzii	positive
GARIN_MALAN	GM85	An.coluzzii	negative
GARIN_MALAN	GM86	An.coluzzii	negative
GARIN_MALAN	GM87	An.coluzzii	negative
GARIN_MALAN	GM89	An.coluzzii	negative
GARIN_MALAN	GM90	An.coluzzii	negative
GARIN_MALAN	GM95	An.coluzzii	negative
GARIN_MALAN	GM96	An.coluzzii	negative
GARIN_MALAN	GM97	An.coluzzii	negative
GARIN_MALAN	GM98	An.coluzzii	negative
GARIN_MALAN	GM99	An.coluzzii	negative
GARIN_MALAN	GM100	An.coluzzii	positive
GARIN_MALAN	GM101	An.coluzzii	negative
GARIN_MALAN	GM102	An.coluzzii	negative
GARIN_MALAN	GM103	An.coluzzii	negative
GARIN_MALAN	GM104	An.coluzzii	positive
GARIN_MALAN	GM105	An.coluzzii	negative
GARIN_MALAN	GM106	An.coluzzii	negative
GARIN_MALAN	GM107	An.coluzzii	negative
GARIN_MALAN	GM108	An.coluzzii	negative
GARIN_MALAN	GM109	An.gambiae	negative
GARIN_MALAN	GM110	An.coluzzii	negative
GARIN_MALAN	GM111	An.coluzzii	negative
GARIN_MALAN	GM113	An.coluzzii	negative
GARIN_MALAN	GM114	An.coluzzii	negative
GARIN_MALAN	GM115	An.coluzzii	negative
GARIN_MALAN	GM116	An.coluzzii	negative
GARIN_MALAN	GM117	An.coluzzii	negative

GARIN_MALAN	GM118	An.coluzzii	negative
GARIN_MALAN	GM119	An.coluzzii	negative
GARIN_MALAN	GM120	An.coluzzii	negative
GARIN_MALAN	GM121	An.coluzzii	negative
GARIN_MALAN	GM122	An.coluzzii	negative
GARIN_MALAN	GM123	An.coluzzii	positive
GARIN_MALAN	GM124	An.coluzzii	negative
GARIN_MALAN	GM125	An.coluzzii	negative

**Plasmodium falciparum Status**
negative
negative
negative
negative
negative
negative
negative
negative
negative
negative
negative
negative
negative
negative
negative
negative
negative
negative
negative
negative
negative
negative
negative
negative
negative
negative
negative
negative
negative
negative
negative
negative
negative
negative
negative
negative
negative
negative
negative
negative
negative
negative
negative
negative
negative
negative
negative
negative
negative

negative
negative
negative
negative
negative
negative
negative
negative
negative
negative
negative
negative
negative
negative
negative
negative
negative
negative
negative
negative
negative
negative
negative
negative
negative
negative
negative
negative
negative
negative
negative
negative
negative
negative
negative
negative
negative
negative
negative
negative
negative
negative
negative
negative
negative
negative
negative
negative
negative
negative

negative
negative
negative
negative
negative
negative
negative
negative
negative
negative
negative
negative
negative
negative
negative
negative
negative
negative
negative
negative
negative
negative
negative
negative
negative
negative
negative
negative
negative
negative
negative
negative
negative
negative
negative
negative
negative
negative
negative
negative
negative
negative
negative
negative
negative
negative
negative
positive
negative
negative

negative
negative
negative
negative
negative
negative
negative
negative
negative
negative
negative
negative
negative
negative
negative
negative
negative
negative
negative
negative
negative
negative
negative
negative
negative
negative
negative
negative
negative
negative
negative
negative
negative
negative
negative
negative
negative
negative
negative
negative
negative
negative
negative
negative
negative
negative
negative
negative
negative
negative

negative
negative
negative
negative
negative
negative
negative
negative
negative
negative
negative
negative
negative
negative
negative
negative
negative
negative
negative
negative
negative
negative
negative
negative
negative
negative
negative
negative
negative
negative
negative
negative
negative
negative
negative
negative
negative
negative
negative
negative
negative
negative
negative
negative
negative
negative
negative
negative
negative
negative

negative
negative
negative
negative
negative
negative
negative
negative
